# Employing a Plant Probiotic Actinomycete for Growth Promotion of Lettuce (*Lactuca sativa* L. var. longifolia) Cultivated in a Hydroponic System under Nutrient Limitation

**DOI:** 10.3390/plants12223793

**Published:** 2023-11-07

**Authors:** Benyapa Kitwetch, Pharada Rangseekaew, Yupa Chromkaew, Wasu Pathom-Aree, Sirasit Srinuanpan

**Affiliations:** 1 Interdisciplinary Program in Biotechnology, Graduate School, Chiang Mai University, Chiang Mai 50200, Thailand; 2 Department of Biology, Faculty of Science, Chiang Mai University, Chiang Mai 50200, Thailand; 3 Center of Excellence in Microbial Diversity and Sustainable Utilization, Faculty of Science, Chiang Mai University, Chiang Mai 50200, Thailand; 4 Department of Plant and Soil Sciences, Faculty of Agriculture, Chiang Mai University, Chiang Mai 50200, Thailand; 5 Biorefinery and Bioprocess Engineering Research Cluster, Chiang Mai University, Chiang Mai 50200, Thailand

**Keywords:** plant growth-promoting actinomycete, hydroponic, lettuce, nutrient limitation, plant probiotic

## Abstract

The consumption of lettuce is associated with an increased risk of ingesting nitrate, a naturally occurring and potentially harmful compound that can have adverse effects on human health. Hydroponic cultivation systems serve as effective tools for regulating nutrient solutions and nitrogen availability, which are essential for controlling nitrate levels. However, the techniques for reducing nutrient levels need to be appropriately calibrated based on lettuce growth responses and their interactions with the environment and growing conditions. Previous studies have demonstrated that plant probiotic actinomycetes can alleviate nutritional stress in various crops. However, there is a noticeable gap in research concerning the effects of actinomycetes on hydroponically grown lettuce, particularly under nutrient-limiting conditions. This study aimed to evaluate the effectiveness of the actinomycete *Streptomyces thermocarboxydus* S3 in enhancing lettuce growth in a nutrient-restricted hydroponic system. The results indicated that the detrimental effects of nutrient stress on lettuce were mitigated by the inoculation of lettuce with *S. thermocarboxydus* S3. This mitigation was evident in various growth parameters, including leaf count, shoot length, and the fresh and dry weights of both shoots and roots. In the presence of nutritional stress, *S. thermocarboxydus* S3 likely mitigated the negative effects on lettuce by reducing hydrogen peroxide levels, presumably through the synthesis of H_2_O_2_-scavenging enzymes. Furthermore, *S. thermocarboxydus* S3 successfully survived and colonized lettuce roots. Therefore, the inoculation of lettuce with *S. thermocarboxydus* S3 offers significant advantages for promoting lettuce growth in nutrient-limited hydroponic systems.

## 1. Introduction

In today’s world, the ever-increasing human population, the quickening pace of urbanization, and rising health concerns have all contributed to the skyrocketing demand for organically grown vegetables, including lettuce. Lettuce, scientifically known as *Lactuca sativa* L., belongs to the Asteraceae family and is widely regarded as one of the most valuable leafy vegetable crops [1]. It is a delectable vegetable enjoyed globally for its crisp texture, pleasant aroma, and rich phytonutrient content, including vitamin C, minerals, and fiber [2]. Numerous studies have demonstrated that lettuce cultivated in hydroponic systems yields large quantities of high-quality produce [1,2]. Hydroponics has emerged as a significant alternative method for lettuce production, owing to several advantages, such as the ease of nutrient control, absence of soil contamination, rapid growth, shorter production cycles, high product quality, and widespread consumer acceptance [3]. Consequently, lettuce is frequently cultivated in hydroponic systems.

There are numerous variables, including fertilizer availability, crop genotype, cultivation techniques, and pest control, that influence the extent to which hydroponic systems can promote optimal plant development, leading to higher yields and better quality [4]. While research has explored lettuce cultivation in hydroponic systems, one of the most costly aspects of hydroponic lettuce production is the need to provide 100 percent of the plant’s fertilizer requirements [5]. In hydroponic lettuce production, the system continuously supplies nutrients to maintain a consistent level of electrical conductivity (EC) for growth promotion [6], resulting in increased production expenses. High concentrations of nutrient solutions, particularly those rich in nitrogen, can be toxic to humans, and leafy lettuces tend to accumulate high levels of nitrates (NO_3_) in their leaves, reducing their nutritional value [5]. If nitrate levels in lettuce exceed the limits established by EU regulations, the product may become less marketable [6]. On the other hand, nutrient deficiency leads to a decrease in the number, size, and resistance of stomata, hindering the growth of hydroponically cultivated lettuce [7]. However, it can prevent the accumulation of excess nutrients or other minerals in the roots and leaves, which might be phytotoxic and have negative consequences when ingested, such as NO_3_ [1]. Therefore, exploring innovative strategies to enhance lettuce development and mitigate the adverse effects of nutrient deficiencies would be beneficial.

To mitigate the adverse effects of abiotic stress on plants, various strategies have been developed, such as the application of exogenous plant growth regulators, like gibberellins, auxins, and cytokinins [8], and the addition of biochar [9,10]. Another approach involves inoculating root-colonizing bacteria into plants, known for their ability to produce plant growth phytohormones [11,12]. Currently, there is growing interest among researchers in microorganisms that stimulate plant growth, driven by environmental concerns and the need for sustainable food production [13]. Actinomycetes, a subgroup of plant growth-promoting bacteria (PGPB), have gained popularity as ‘plant probiotics’ and valuable tools for enhancing crop yields through environmentally friendly agriculture practices [14,15]. Actinomycetes have demonstrated their ability to stimulate the growth of various plants under both normal and stressful conditions [14,16,17]. They achieve this through multiple mechanisms, including the production of phytohormones and siderophores, as well as phosphate solubilization [16]. For instance, specific actinomycete strains, such as *Streptomyces* sp. HM2, *Streptomyces thinghirensis* HM3, *Streptomyces* sp. HM8, and *S. tricolor* HM10, have demonstrated the potential to enhance nutrient availability, generate phytohormones, and promote cucumber growth even under abiotic stress conditions [15]. Other plants, including rice [14] and tomatoes [18], have also benefited from *Streptomyces* spp. in terms of growth enhancement. Beyond actinomycetes, non-actinomycete microorganisms, such as *Gluconacetobacter diazotrophicus*, *Rhizobium laguerreae*, and *Bacillus* spp., have shown the ability to enhance the growth of hydroponically cultivated lettuce [1,5,19]. However, there remains a lack of research and practical utilization of actinomycetes, especially *Streptomyces* strains, in hydroponically grown plants facing nutritional stress.

Therefore, this research aimed to determine how well a plant probiotic actinomycete performed in boosting the growth of lettuce in a nutrient-restricted hydroponic system. Specifically, *S. thermocarboxydus* S3 was chosen because it shows great promise as a PGPB [14] under abiotic stress and also has the ability to fix nitrogen, but no research has been conducted on the effectiveness of this strain in a nutrient-limiting hydroponic system for growing lettuce.

## 2. Materials and Methods

### 2.1. Microorganism and Spore Preparation

Actinomycete *Streptomyces thermocarboxydus* S3, obtained from the Division of Microbiology, Department of Biology, Faculty of Science, Chiang Mai University, was used as a plant probiotic strain. *S. thermocarboxydus* S3 was cultured on ISP2 agar containing 10 g/L of malt extract, 4 g/L of yeast extract, 4 g/L of glucose, and 15 g/L of agar at 37 ± 1 °C for 10 days to stimulate the spore germination. After 10 days of cultivation, the spore suspension of *S. thermocarboxydus* S3 was prepared by adding sterilized deionized water to cultured plates. The spore suspension was then diluted to the appropriate concentration to obtain an optical density (OD_600_ nm) of 1.00, which is equal to 10^8^ spores/mL. The spore suspension at 10^8^ spores/mL was used in all experiments in this study.

### 2.2. Inoculant Preparation

Lettuce (*Lactuca sativa* L. var. longifolia) seeds obtained from the Vegetable Seed Production and Organic Farming Learning Center, Maejo University, Thailand, were first sterilized using 70% *v/v* ethanol for 1 min, soaked in 1.2% *v/v* NaClO solution for 12 min, and finally washed with sterilized deionized water three times for 1 min. The surface-sterilized lettuce seeds were incubated on nutrient agar containing 5 g/L of peptone, 5 g/L of NaCl, 2 g/L of yeast extract, 1 g/L of beef extract, and 20 g/L of agar at 37 ± 1 °C for 2 days to confirm the completeness of surface sterilization. Thirty surface-sterilized lettuce seeds were sown on Petri plates with wet filter paper at room temperature (30 ± 1 °C). After 7 days, the germination percentage was recorded from number of germinated seeds compared with the first day. Then, the surface-sterilized lettuce seeds were inoculated with *S. thermocarboxydus* S3 spore suspension at 10^8^ spores/mL and/or sterilized deionized water on a shaker with a shaking speed of 120 rpm at room temperature for 3 h before being sown in the growing tray.

### 2.3. Experimental Design

**Figure 1:**
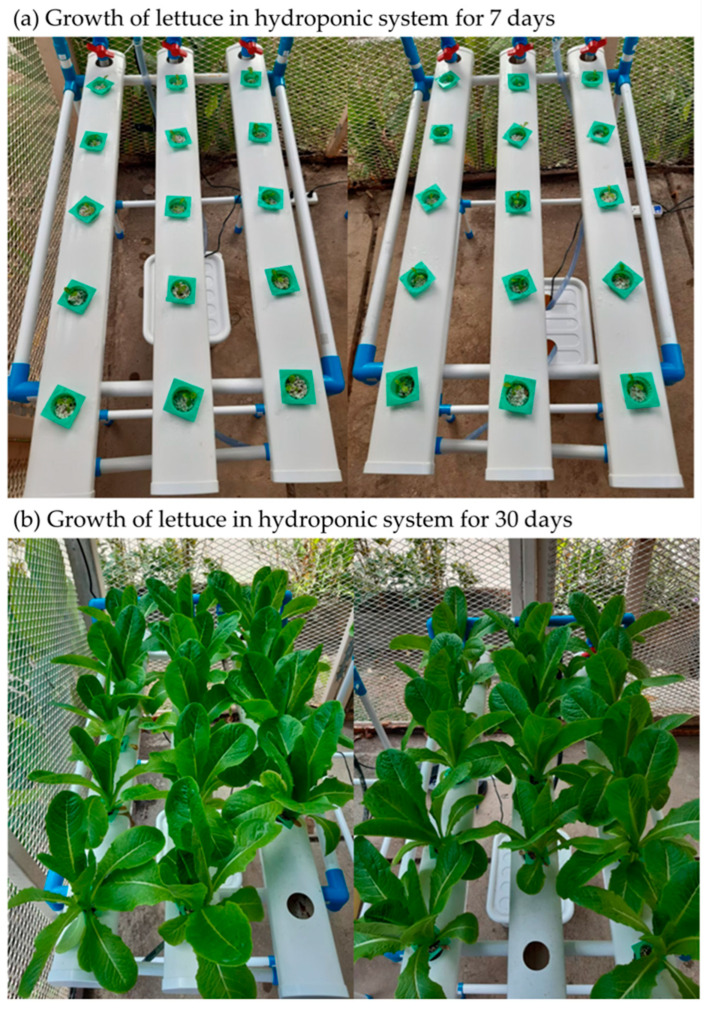
Growing lettuce in the nutrient film technique (NFT) system after 7 days (**a**) and 30 days (**b**).

Both inoculated and uninoculated lettuce seeds were sown in the growing tray containing a mixture of perlite and vermiculite at a ratio of 3:1, placed under greenhouse conditions (12 h photoperiod, 306–2311 µmol m^−2^ s^−1^ photosynthetically active radiation, 20–27 °C temperature range, and 61–76% relative humidity range), and watered daily. Lettuce was transplanted into the nutrient film technique (NFT) system after 7 days of seedling growth (when the plants were approximately 5 cm in length with three to four true leaves). The system consisted of several different parts, including tanks with a capacity of 30 L each (for the nutrient solutions), a modest pump that was used to power the system, perlite media that was used to support the plant, and one-meter-tall pots that had pipes and micropipes connected to the base of the pots (Figure 1). There are three pots, each of which contains five plants (equally 15 plants/hydroponic system), and tanks containing nutritional solutions that are linked to the pots by pipes and micropipes. This research used a 4-unit hydroponic system based on the treatments mentioned below.

The hydroponic nutrient solution was obtained from Kitsuwan Farm, Notaburi, Thailand. Solution A (57.5 g/L of Ca(NO_3_)_2_, 1 g/L of 7% Fe-DTPA, and 2 g/L of 13.2% Fe-EDTA) and solution B (30 g/L of KNO_3_, 25 g/L of MgSO_4_, 13.25 g/L of KH_2_PO_4_, 2.5 g/L of Dissolvine^®^ ABC EDTA, 0.5 g/L of 13% Mn-EDTA) were combined in accordance with the directions provided by the manufacturer. The full-strength nutrient solution contained an electrical conductivity (EC) of 900 µS/cm. The experiment was conducted with the following treatments: (i) inoculated seeds + full-strength nutrient solution, (ii) uninoculated seeds + full-strength nutrient solution, (iii) inoculated seeds + half-strength nutrient solution, and (iv) uninoculated seeds + half-strength nutrient solution. EC values were maintained at 900 µS/cm for full-strength nutrient solution and 450 µS/cm nutrient solution for half-strength nutrient solution. Every other day, the EC value in all treatments was measured and maintained at the specified value for each treatment throughout the experiment by adding the stock solution accordingly.

### 2.4. Analytical Methods

After 30 days of cultivation, the lettuce was harvested, and the following parameters were measured: leaf number, shoot and root length, shoot and root fresh weight, and shoot and root dry weight after drying in an oven at 70 °C until constant weight.

For the determination of chlorophylls and carotenoids, one gram of fresh leaves was mashed and mixed with 80% *v/v* of acetone. The mixture was filtered through Whatman paper No. 1 and then centrifuged at 5000 rpm for 10 min to obtain the supernatant. The supernatant was spectrophotometrically measured at absorbances of 480, 645, and 663 nm. The concentrations of chlorophyll *a*, chlorophyll *b*, total chlorophyll, and carotenoids were calculated using the equations reported by Arnon [20].

For the determination of hydrogen peroxide content, 100 mg of fresh leaves were mashed and mixed with 3 mL of 0.1% *w/v* TCA and then centrifuged at 10,000 rpm for 15 min at 4 °C to obtain the supernatant. The supernatant (0.5 mL) was mixed with 0.5 mL of 10 mM potassium phosphate buffer (pH 7) and 1 mL of 1 M potassium iodide. The mixture was spectrophotometrically measured at an absorbance of 390 nm. The hydrogen peroxide content was calculated using the equations reported by Velikova et al. [21].

Colonization of lettuce root by *S. thermocarboxydus* S3 was confirmed by dilution spread plate and microscopic examination. Briefly, the sterilized root was ground in sterile distilled water. The root suspension (1 mL) was serially diluted and spread on ISP2 agar that was supplemented with 0.1 mg/mL nalidixic acid and 0.1 mg/mL cycloheximide. The suspension was then incubated at room temperature for 14 days. The putative actinomycete colonies were isolated, re-streaked on ISP2 agar, and morphologically compared with the purified *S. thermocarboxydus* S3. The 16S rRNA gene sequencing analysis was also performed to confirm the identity of the obtained isolate, according to the report of Lasudee et al. [14]. The 16S rRNA gene sequences were compared with closely related sequences in the GenBank database. To visualize the colonization of *S. thermocarboxydus* S3 in lettuce roots, the sterilized roots were submerged in 10% KOH for 5 m, immersed in 1% HCl for 24 h, and then washed with sterile distilled water. After that, roots were stained in 0.06% methyl blue before microscopic observation [22].

### 2.5. Statistical Analysis

Each experiment was performed with three independent replicates, and the results are reported as the mean value ± standard deviation. A one-way analysis of variance (ANOVA) and Duncan’s multiple range tests were used in order to determine whether the results of the experiment were statistically significant (*p*-value < 0.05).

## 3. Results

**Figure 2:**
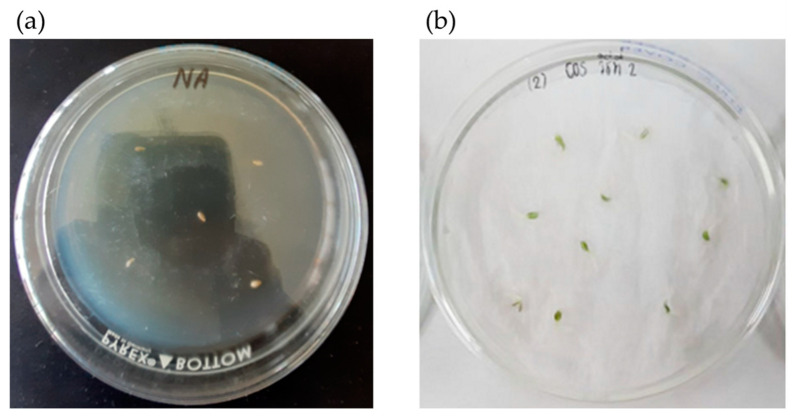
Effect of surface sterilization on lettuce seeds. (**a**) Sterility check by incubating surface-sterilized lettuce seeds on NA media at 37 °C for 72 h; and (**b**) determination of seed germination after sowing surface-sterilized lettuce seeds on wet filter paper in Petri dishes at room temperature for 7 days.

The effect of the surface sterilization method on lettuce seeds was investigated, and the findings of incubating surface-sterilized lettuce seeds on nutrient agar (NA) media at 37 °C for 72 h showed no microbial growth on the plates (Figure 2a). Seven days after sowing surface-sterilized lettuce seeds on wet filter paper in Petri dishes at room temperature, the germination rate was determined to be 96.67% (Figure 2b). This demonstrated that the sterilization method used in this study had no effect on lettuce growth and can be used to remove native microorganisms prior to inoculation with the plant probiotic actinomycete.

**Figure 3:**
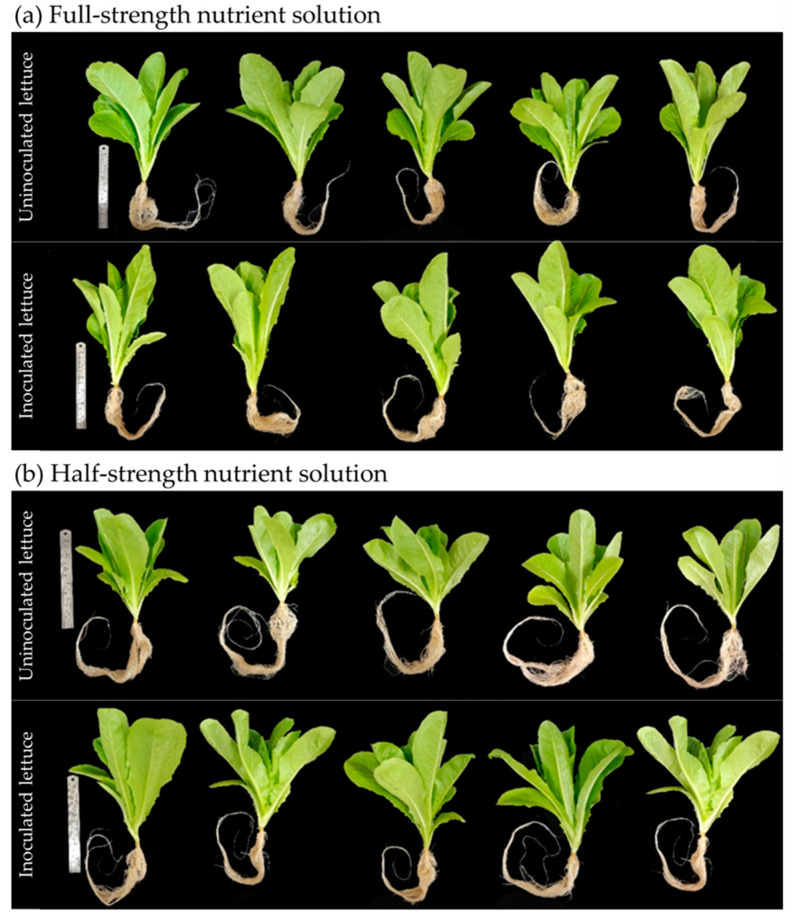
Growth of hydroponic lettuce inoculated and uninoculated with *Streptomyces thermocarboxydus* S3 under (**a**) full-strength nutrient solution at an EC of 900 µS/cm and (**b**) half-strength nutrient solution at an EC of 450 µS/cm.

**Figure 4:**
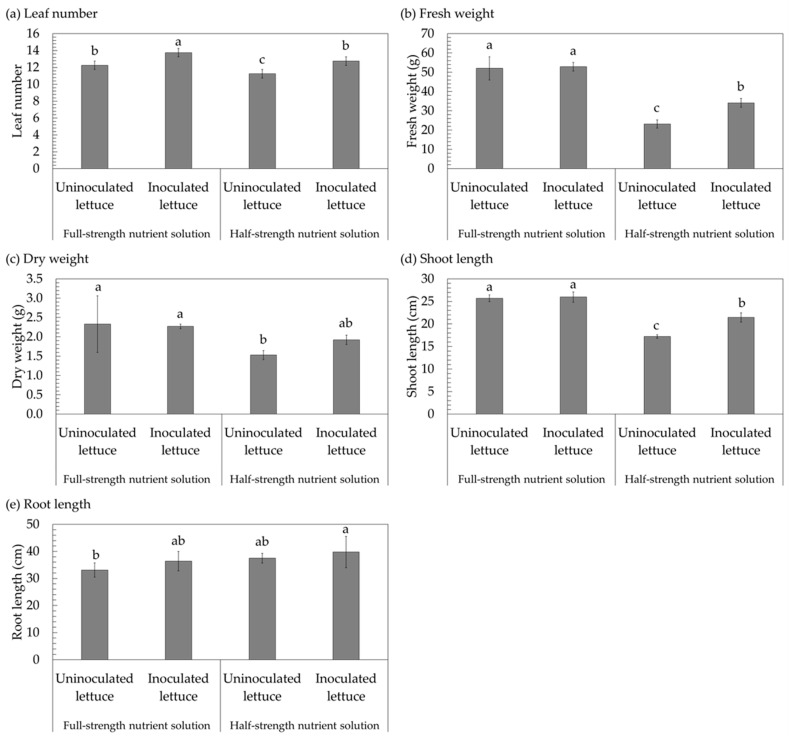
Growth promotion (leaf number (**a**), fresh weight (**b**), dry weight (**c**), shoot length (**d**), and root length (**e**)) of lettuce inoculated and uninoculated with *Streptomyces thermocarboxydus* S3 cultivated in a hydroponic system under full-strength nutrient solution at an EC of 900 µS/cm and half-strength nutrient solution at an EC of 450 µS/cm. Different lowercase letters (a, b, or c) indicate significant differences among the treatments (*p* < 0.05).

The inoculation effects of the probiotic actinomycete *Streptomyces thermocarboxydus* S3 were investigated in lettuce that was grown in a hydroponic system with either full- or half-strength nutrient solution. The leaf number, shoot and root length, shoot and root fresh weight, and shoot and root dry weight were recorded after 30 days of lettuce cultivation. The results found that with full-strength nutrient solution, there was no visible difference in the physical traits between inoculated and uninoculated lettuce (Figure 3a), while a comparison of the growth of inoculated and uninoculated lettuce cultured in half-strength nutrient solution revealed that uninoculated lettuce had poorer growth (Figure 3b). The number of lettuce leaves on inoculated lettuce was 13.40 with a half-strength nutrient solution, which was slightly higher than the number of lettuce leaves on uninoculated lettuce (12.40 leaves) (Figure 4a and Table 1). The number of leaves on inoculated lettuce cultivated in a half-strength nutrient solution was 12.40, which was significantly greater than the number of leaves on uninoculated lettuce, which was 10.80 (Figure 4a and Table 1).

Considering the shoot and root fresh weight, it was found that the shoot and root fresh weight of inoculated lettuce (51.95 g) with a full-strength nutrient solution was slightly greater than that of uninoculated lettuce (49.01 g) (Figure 4b and Table 1). However, when lettuce was grown in a half-strength nutrient solution, the fresh weight of the shoots and roots was reduced by about 1.6–2.3 times compared to when lettuce was grown in a full-strength nutrient solution. With a half-strength nutrient solution, the fresh weight of the shoots and roots was greater in the inoculated lettuce (31.97 g) compared to lettuce that was not inoculated (21.58 g) (Figure 4b and Table 1). Moreover, the trend shown in the shoot and root fresh weight of lettuce was mirrored by the trend seen in the shoot and root dry weight of lettuce. According to Figure 4c and Table 1, it was observed that the shoot and root dry weight of lettuce grown in a full-strength nutrient solution was greater than that of lettuce grown in a half-strength nutrient solution. The dry weight of inoculated lettuce likewise exceeded that of uninoculated lettuce. About 2.01 g and 2.33 g of shoot and root dry weight were recorded for uninoculated and inoculated lettuce, respectively, grown in full-strength nutrient solution, while shoot and root dry weights of uninoculated and inoculated lettuce were recorded at 1.43 g and 1.82 g, respectively, for half-strength nutrient solution (Figure 4c and Table 1).

Regarding shoot and root lengths, the shoot length of inoculated lettuce (25.24 cm) was comparable to that of uninoculated lettuce (25.70 cm) when grown in full-strength nutrient solution; however, when grown in half-strength nutrient solution, the shoot length of inoculated lettuce (21.90 cm) was significantly longer than that of uninoculated lettuce (16.66 cm) (Figure 4d and Table 1). Interestingly, when lettuce was grown in half-strength nutrient solution, the root length was much longer than when lettuce was cultivated in full-strength nutrient solution. Both half- and full-strength nutrient solutions resulted in a larger root length for inoculated lettuce (37.82 cm for full-strength nutrient solution and 43.32 cm for half-strength nutrient solution) as compared to uninoculated lettuce (34.90 cm for full-strength nutrient solution and 36.76 cm for half-strength nutrient solution) (Figure 4e and Table 1).

**Figure 5:**
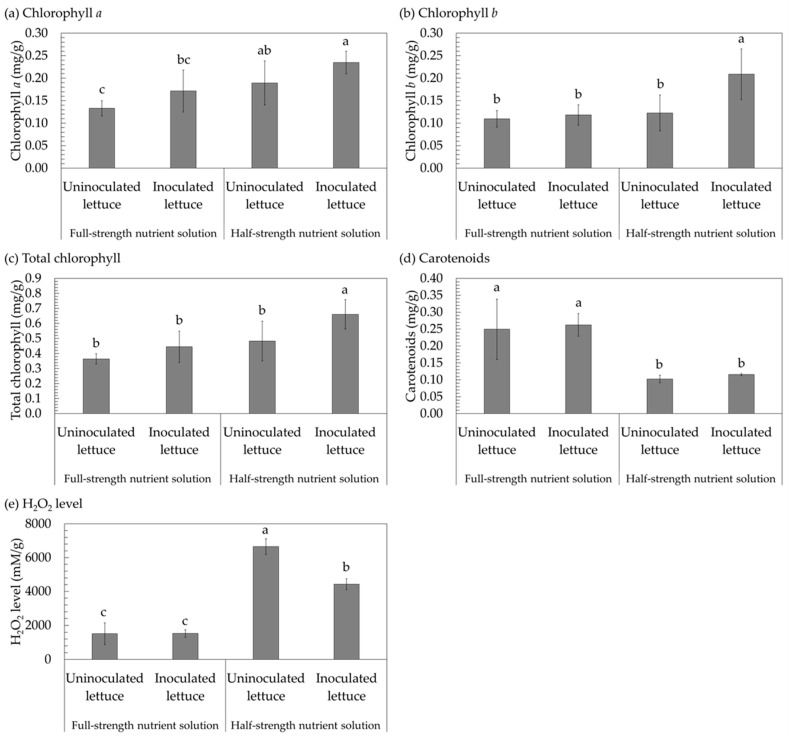
Biochemical parameters (chlorophyll *a* (**a**), chlorophyll *b* (**b**), total chlorophyll (**c**), carotenoids (**d**), and H_2_O_2_ production (**e**)) of lettuce inoculated and uninoculated with *Streptomyces thermocarboxydus* S3 cultivated in a hydroponic system under full-strength nutrient solution at an EC of 900 µS/cm and half-strength nutrient solution at an EC of 450 µS/cm. Different lowercase letters (a, b, or c) indicate significant differences among the treatments (*p* < 0.05).

Chlorophyll *a*, chlorophyll *b*, total chlorophyll content, carotenoids, and hydrogen peroxide (H_2_O_2_) concentration were examined to determine biochemical changes in lettuce after inoculation with *S. thermocarboxydus* S3 under nutritional restriction (Figure 5 and Table 1). When compared to uninoculated lettuce, inoculated lettuce had considerably greater levels of chlorophyll *a* (Figure 5a), chlorophyll *b* (Figure 5b), total chlorophyll (Figure 5c), and carotenoids (Figure 5d). The chlorophyll *a*, chlorophyll *b*, and total chlorophyll levels produced by the half-strength nutrient solution were greater than those produced by the full-strength nutrient solution. However, the amount of carotenoids found in lettuce cultivated with the half-strength nutrient solution was lower than that found in lettuce grown with the full-strength nutrient solution (Figure 5d and Table 1). Under the full-strength nutrient solution, the H_2_O_2_ concentration of inoculated lettuce was similar to that of uninoculated lettuce; however, when grown in the half-strength nutrient solution, the H_2_O_2_ concentration was significantly higher than that of uninoculated lettuce (Figure 5e and Table 1).

**Figure 6:**
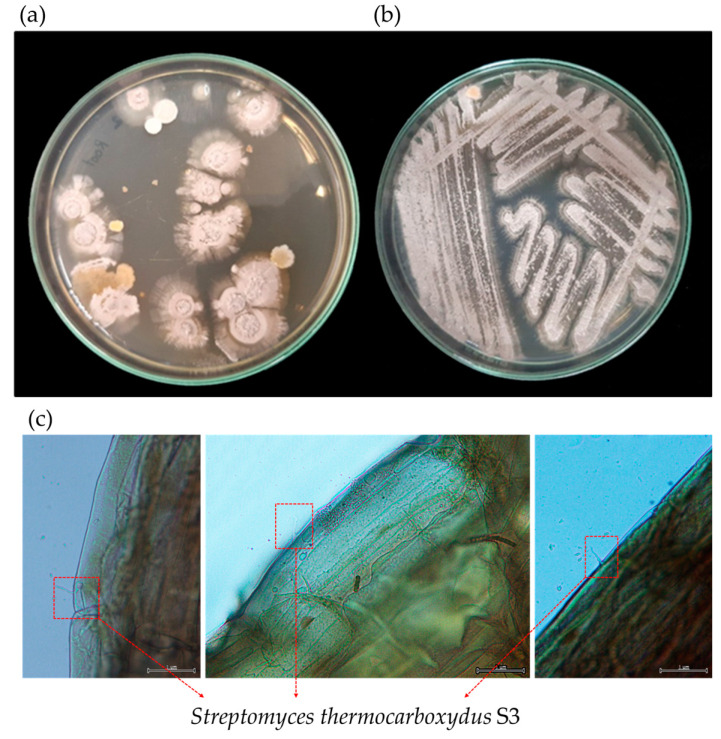
Colonization of *Streptomyces thermocarboxydus* S3 in lettuce roots using a culture-based method (colony characteristics of re-isolated *S. thermocarboxydus* S3 from lettuce roots (**a**) and pure culture of re-isolated *S. thermocarboxydus* S3 (**b**)) and light microscopic examination (**c**).

From the inoculated lettuce roots, a total of 1.2 × 10^4^ CFU/mL of *S. thermocarboxydus* S3 was re-isolated (Figure 6a,b). The validation of putative *S. thermocarboxydus* S3, which was carried out by 16S rRNA gene sequencing (see Supplementary Materials), indicated a similarity of 97.5% with *S. thermocarboxydus* DSM 44293^T^. The colonization of *S. thermocarboxydus* S3 in the roots of lettuce was verified by dye-staining examination. A microscopic image revealed the characteristic filamentous cells of *S. thermocarboxydus* S3 on the root tissue (Figure 6c).

## 4. Discussion

Plant probiotic actinomycetes are well-recognized plant growth-promoting bacteria. They stimulate plant development via a variety of methods, both directly and indirectly, which contribute to plant growth [17], resulting in improved crop yield and cost reduction. Members of the genus *Streptomyces*, which are classified as actinomycetes, are among the most prevalent soil bacteria and are widely distributed in the rhizosphere [23]. It is widely established that these bacteria are promising candidates for promoting the development of numerous plants, including rice, lettuce, tomato, and cucumber, among others [14,15,18,24]. The production of phytohormones, the solubilization of inorganic phosphate, the production of siderophores that chelate iron from the environment, and the inhibition of phytopathogens are all strategies that *Streptomyces* uses to promote and increase plant development [14]. The present study was designed to apply the plant growth-promoting actinomycete *Streptomyces thermocarboxydus* S3 in an attempt to investigate its potential to promote the growth of lettuce (*Lactuca sativa* L. var. longifolia) in a hydroponic system under conditions of nutrient stress. We employed *S. thermocarboxydus* S3 given its ability to increase yields of Thai jasmine rice grown in low-nutrient soil and subjected to artificial drought and its capacity to synthesize indole-3-acetic acid (IAA), siderophores, and phosphate solubility [14]. The presence of these plant-growth-stimulating traits in *S. thermocarboxydus* S3 lends credence to the prospect of employing this actinomycete for agricultural uses, particularly in regions that are subject to stress caused by a nutrient deficiency.

*Streptomyces* has been shown to have a positive impact on lettuce’s growth and development, with some studies showing a considerable increase in vegetative growth trials [24,25], but there is no information available on the use of *S. thermocarboxydus* S3 in the process of encouraging the development of lettuce in a hydroponic system. The need to provide 100 percent of the required fertilizer for hydroponic lettuce cultivation is one of the factors that increases production costs. By lowering the concentration of the nutrient solution and then inoculating lettuce with *S. thermocarboxydus* S3, it is feasible to reduce the overall cost of production. The benefit of *S. thermocarboxydus* S3 inoculation is that it secretes phytohormones as well as organic acids, both of which help promote microelements in hydroponic systems. In the present study, the results revealed an increase in the number of leaves, shoot length, the fresh weight of the shoot and root, as well as the dry weight of the shoot and roots in inoculated lettuce when compared to uninoculated lettuce. However, a trial with lower nutrient content resulted in lower yields than those obtained with a greater nutritional concentration. This indicated that at these lower levels, key nutrients, like nitrogen, would be in short supply. Although *S. thermocarboxydus* S3 is capable of producing plant growth agents, there are not enough quantities for them to be sufficient in hydroponic systems under lower nutrient concentrations. These findings are consistent with those found by Sebring et al. [5], who demonstrated that at a reduced nitrogen content and when *Gluconacetobacter diazotrophicus* was used with two lettuce cultivars under hydroponic circumstances, there was a substantial decrease in most lettuce production characteristics.

The nutritional solution concentration was noticeably reduced at the longer lettuce root lengths, and lettuce inoculated with *S. thermocarboxydus* S3 exhibited marginal root growth extension. Similarly, in response to nutritional deficiency, there was an increase in root length in kauri, kahikatea, rewarewa, and pukatea [26]. It was shown that poor fertilizer concentration led to the longest roots [27]. In general, when there is a lack of uniformity in the availability of nitrogen, lateral roots spread out towards high-N areas, and this sensitivity to N-foraging has been thoroughly investigated for nitrate (NO_3_^−^) [28]. Interestingly, Oldroyd and Leyser [27] mentioned that a rhizobial bacteria association affects root length under nutrient-deficient conditions because these microbes are already functioning as a branch of the plant’s root system. This suggests that *S. thermocarboxydus* S3 root colonization may have played a role in the modest increase in root length seen in inoculated lettuce. However, in hydroponically grown lettuce, root length is irrelevant.

The increase in photosynthetic pigments may help capture and use light more efficiently by reflecting the light energy that is absorbed, transmitted, dispersed, and spread by leaves. This has an immediate impact on both the rate of photosynthesis and the rate of growth, which, in the end, has an impact on yield and the efficiency with which light is used [29]. In the present study, the leaf chlorophyll *a*, chlorophyll *b*, and total chlorophyll concentrations of both inoculated and uninoculated lettuce increased in a quadratic trend as the nutrient levels decreased. This demonstrates that, as a means of adapting to nutritional constraints, particularly low-N stress, lettuce may improve its capacity to gather light by expanding the size of its light-harvesting complex. This will ultimately lead to increased chlorophyll production. Similarly, Wu et al. [30] found that the maize plant was able to absorb and transmit more light energy in its leaves after being subjected to stress caused by low levels of nitrogen. This led to an increase in photosynthesis as well as chlorophyll production. In contrast, the overall amount of carotenoids was lower as the nutritional solution became less concentrated. Because of the limited availability of nutrients, plants must maintain a higher chlorophyll content than carotenoids in order to continue growth and development. This is necessary for the process of photosynthesis, which is essential for plant survival. Chlorophyll is responsible for setting in motion a chain of electron transfer reactions that ultimately results in the production of carbohydrates from carbon dioxide. In contrast, carotenoids are unable to transport absorbed light into the photosynthetic pathway in a direct way; nevertheless, they may transfer their light to chlorophylls, which assist in the process of photosynthesis [31].

Lettuce inoculated with *S. thermocarboxydus* S3 had higher amounts of photosynthetic pigments than non-inoculated lettuce. Studies have shown that the production of carotenoids is enhanced when growth-promoting microbes, such as phosphate solubilizers, colonize plant roots [32]. Furthermore, microorganisms have the ability to influence the biosynthesis of growth-promoting substances as well as other molecules, such as auxins, which also have a favorable impact on the creation of photosynthetic pigments [33]. Some growth-associated compounds, such as cytokinins [34], are influenced by phosphate-solubilizing bacteria, which have a beneficial impact on chlorophyll production [29]. Reis et al. [35] demonstrated that soybeans inoculated with *Paenibacillus alvei* and/or *Lysinibacillus fusiformis* increase biomass and chlorophyll synthesis. Therefore, in the present study, the ability of *S. thermocarboxydus* S3 to act as a phosphate solubilizer and phytohormone producer [14] may explain the observed effects on the concentration of photosynthetic pigments in lettuce that was inoculated with this strain.

The oxidative stress caused by an inadequate supply of nutrients results in the production of reactive oxygen species (ROS) in plants [36]. When ROS accumulate at excessive levels, they block enzymes, oxidize lipids and proteins, damage DNA and RNA, and activate cell death mechanisms [17]. The most significant nonradical ROS is hydrogen peroxide (H_2_O_2_), which also serves as a reliable indicator of oxidative stress [16]. In the present study, oxidative stress occurred as a result of the limitation in nutrients since the H_2_O_2_ level was higher in the half-strength nutrient solution than in the full-strength nutrient solution. Lettuce inoculated with *S. thermocarboxydus* S3 exhibited a level of H_2_O_2_ in its leaves that was comparable to the amount obtained in uninoculated lettuce plants that were grown in full-strength nutrient solutions. This finding indicates that both *S. thermocarboxydus* S3-inoculated and uninoculated lettuces were subjected to identical levels of stress. Interestingly, when lettuce was inoculated with *S. thermocarboxydus* S3, the levels of H_2_O_2_ in the leaves were less those of uninoculated lettuce when grown in the half-strength nutrient solution. It is possible that *S. thermocarboxydus* S3 could improve H_2_O_2_-scavenging enzymes, such as catalase (CAT) and peroxidase (POD). These enzymes aid in the maintenance of H_2_O_2_ homeostasis and contribute to plant development under conditions of nutritional stress. In most cases, the elimination of ROS may be accomplished by either enzymatic or non-enzymatic antioxidative systems. Since *S. thermocarboxydus* is capable of producing both CAT [37] and POD [38], it is possible that *S. thermocarboxydus* isolate S3 also produces both enzymes in order to scavenge H_2_O_2_. Previously, when tomatoes were subjected to biotic stress, inoculation with deep-sea *Dermacoccus* decreased the levels of H_2_O_2_ while simultaneously increasing CAT and POD [16]. The reduction in H_2_O_2_ and superoxide dismutase in salt-stressed mung bean leaves caused by inoculation with *Bacillus cereus* was accompanied by an increase in the activities of CAT and POD in the leaves [39]. Therefore, it should be noted that the inoculation of lettuce with *S. thermocarboxydus* S3 was responsible for the reduction in H_2_O_2_ generation as one strategy for protecting plants from the negative effects of nutritional stress.

A culture-based method and a dye-staining investigation both demonstrated that *S. thermocarboxydus* S3 was able to colonize lettuce roots and that it was able to survive in this environment. It has been hypothesized that this particular isolate is able to colonize the roots of lettuce due to the fact that *S. thermocarboxydus* S3 was successfully re-isolated from the roots as well as the detection of the characteristic filamentous cells of *Streptomyces* on the root surface. In addition, significant numbers of *S. thermocarboxydus* S3 were recovered from harvested roots, indicating that this strain had been able to live inside the root tissues. Previous research has shown evidence of bacterial colonization that is comparable to our findings [14,17]. In addition to facilitating plant development, bacterial colonization of plant roots is crucial to their survival under abiotic stresses, such as nutrient constraints [27].

Although the inoculation of lettuce with *S. thermocarboxydus* S3 offers significant advantages for promoting lettuce growth in nutrient-limited hydroponic systems, further research and experimentation are necessary to investigate other mechanisms influenced by nutrient limitations. This includes examining nutrient contents, related gene expression, and the response of actinomycetes to varying nutrient concentrations. These efforts might achieve a more comprehensive understanding of the mechanisms underlying actinomycetes’ assistance in nutrient-deficient conditions.

## 5. Conclusions

Our results suggest that the plant probiotic actinomycete *Streptomyces thermocarboxydus* S3 can enhance the growth of lettuce (*Lactuca sativa* L. var. longifolia) when cultivated in a hydroponic system. Inoculating lettuce with *S. thermocarboxydus* S3 mitigated the detrimental effects of nutrient stress, as evidenced by various growth metrics, including leaf count, shoot length, and the fresh and dry weights of both shoots and roots. When nutritional stress was present, *S. thermocarboxydus* S3 appeared to mitigate the negative effects on lettuce by reducing hydrogen peroxide levels, presumably through the synthesis of H_2_O_2_-scavenging enzymes. Both a culture-based method and dye-staining examination confirmed the survival and colonization of lettuce roots by *S. thermocarboxydus* S3. Our study highlights the promising potential of *S. thermocarboxydus* S3 as a plant probiotic bacterium for the development of a bioinoculant. Such an inoculant could be valuable for facilitating the long-term, environmentally friendly management of hydroponic plants facing nutrient deficiency.

## Figures and Tables

**Table 1:** Growth and biochemical parameters of lettuce inoculated and uninoculated with *Streptomyces thermocarboxydus* S3 cultivated in a hydroponic system under full-strength nutrient solution at an EC of 900 µS/cm and half-strength nutrient solution at an EC of 450 µS/cm.

Parameters	Full-Strength Nutrient Solution		Half-Strength Nutrient Solution	
	Uninoculated Lettuce	Inoculated Lettuce	Uninoculated Lettuce	Inoculated Lettuce
Number of leaves	12.25 ± 0.50 ^b^	13.75 ± 0.50 ^a^	11.25 ± 0.50 ^c^	12.75 ± 0.50 ^b^
Fresh weight (g)	52.01 ± 6.00 ^a^	52.86 ± 2.23 ^a^	23.12 ± 2.12 ^c^	34.11 ± 2.30 ^b^
Dry weight (g)	2.33 ± 0.73 ^a^	2.27 ± 0.06 ^a^	1.53 ± 0.12 ^b^	1.92 ± 0.12 ^ab^
Root length (cm)	33.10 ± 2.63 ^b^	36.38 ± 3.59 ^ab^	37.48 ± 1.78 ^ab^	39.75 ± 5.79 ^a^
Shoot length (cm)	25.70 ± 0.77 ^a^	25.98 ± 1.15 ^a^	17.18 ± 0.39 ^c^	21.45 ± 1.05 ^b^
Chlorophyll *a* (mg/g)	0.1329 ± 0.0169 ^c^	0.1715 ± 0.0465 ^bc^	0.1895 ± 0.0489 ^ab^	0.2348 ± 0.0250 ^a^
Chlorophyll *b* (mg/g)	0.1096 ± 0.0183 ^b^	0.1182 ± 0.0226 ^b^	0.1227 ± 0.0399 ^b^	0.2089 ± 0.0563 ^a^
Total chlorophyll (mg/g)	0.3646 ± 0.0343 ^b^	0.4450 ± 0.1040 ^b^	0.4829 ± 1318 ^b^	0.6608 ± 0.0976 ^a^
Carotenoids (mg/g)	0.2493 ± 0.0890 ^a^	0.2625 ± 0.0339 ^a^	0.1025 ± 0.0112 ^b^	0.1157 ± 0.0036 ^b^
H_2_O_2_ (mM/g)	1515.63 ± 640.91 ^c^	1528.13 ± 220.41 ^c^	6650.00 ± 460.19 ^a^	4428.13 ± 325.54 ^b^

Noted: All values are the mean ± SD. The values in each row are indicated by letters (a, b, or c) that are significantly (*p* < 0.05) different from each other based on Duncan’s new multiple range test.

## Data Availability

Data will be made available on request.

## Supplementary Materials

The following supporting information can be downloaded at: https://www.mdpi.com/article/10.3390/plants12223793/s1, Table S1: 16S rRNA sequence of isolated actinomycete.
