# Supplementary material for: Employing a Plant Probiotic Actinomycete for Growth Promotion of Lettuce (Lactuca sativa L. var. longifolia) Cultivated in a Hydroponic System under Nutrient Limitation
Source: Plants (Basel). 2023 Nov 7;12(22):3793. doi: 10.3390/plants12223793 (PMC10675278; doi:10.3390/plants12223793)
Supplement: Supplementary file 1 [file plants-12-03793-s001.zip › plants-2638193-supplementary.pdf]

Supplementary materials

Employing a Plant Probiotic Actinomycete for Growth Promotion of Lettuce (*Lactuca sativa* L. var. longifolia) Cultivated in a Hydroponic System under Nutrient Limitation

Benyapa Kitwetch [1], Pharada Rangseekaew [2,3], Yupa Chromkaew [4], Wasu Pathom-Aree [2,3,*] and Sirasit Srinuanpan [2,3,5,*]

[1] Interdisciplinary Program in Biotechnology, Graduate School, Chiang Mai University, Chiang Mai 50200, Thailand
[2] Department of Biology, Faculty of Science, Chiang Mai University, Chiang Mai 50200, Thailand
[3] Center of Excellence in Microbial Diversity and Sustainable Utilization, Faculty of Science, Chiang Mai University, Chiang Mai 50200, Thailand
[4] Department of Plant and Soil Sciences, Faculty of Agriculture, Chiang Mai University, Chiang Mai 50200, Thailand;
[5] Biorefinery and Bioprocess Engineering Research Cluster, Chiang Mai University, Chiang Mai 50200, Thailand
* Correspondence: wasu.p@cmu.ac.th (W.P.-a); sirasit.s@cmu.ac.th (S.S.)

Table S1. 16S rRNA sequence of isolated actinomycete

16S rRNA Sequence
TTCGGGGTGGATTAGTGGCGAACGGGTGAGTAACACGTGGGCAATCTGCC<br>CTGCACTCTGGGACAAGCCCTGGAAACGGGGTCTAATACCGGATACTGAT<br>CGCCTTGGGCATCCTTGGTGATCGAAAGCTCCGGCGGTGCAGGATGAGCC<br>CGCGGCCTATCAGCTTGTTGGTGAGGTAATGGCTCACCAAGGCGACGACG<br>GGTAGCCGGCCTGAGAGGGCGACCGGCCACACTGGGACTGAGACACGGC<br>CCAGACTCCTACGGGAGGCAGCAGTGGGGAATATTGCACAATGGGCGAA<br>AGCCTGATGCAGCGACGCCGCGTGAGGGATGACGGCCTTCGGGTTGTAAA<br>CCTCTTTCAGCAGGGAAGAAGCGAAAGTGACGGTACCTGCAGAAGAAGC<br>GCCGGCTAACTACGTGCCAGCAGCCGCGGTAATACGTAGGGCGCGAGCGT<br>TGTCCGGAATTATTGGGCGTAAAGAGCTCGTAGGCGGCTTGTCGCGTCGGT<br>TGTGAAAGCCCGGGGCTTAACCCCGGGTCTGCAGTCGATACGGGCAGGCT<br>AGAGTTCGGTAGGGGAGATCGGAATTCCTGGTGTAGCGGTGAAATGCGCA<br>GATATCAGGAGGAACACCGGTGGCGAAGGCGGATCTCTGGGCCGATACTG<br>ACGCTGAGGAGCGAAAGCGTGGGGAGCGAACAGGATTAGATACCCTGGT<br>AGTCCACGCCGTAAACGGTGGGCACTAGGTGTGGGCGACATTCCACGTCG<br>TCCGTGCCGCAGCTAACGCATTAAGTGCCCCGCCTGGGGAGTACGGCCGC<br>AAGGCTAAAACTCAAAGGAATTGACGGGGGCCCGCACAAGCGGCGGAGC<br>ATGTGGCTTAATTCGACGCAACGCGAAGAACCTTACCAAGGCTTGACATA<br>CACCGGAAACGTCCAGAGATGGGCGCCCCCTTGTGGTCGGTGTACAGGTG<br>GTGCATGGCTGTCGTCAGCTCGTGTCGTGAGATGTTGGGTTAAGTCCCGCA<br>ACGAGCGCAACCCTTGTCCCGTGTTGCCAGCAGGCCCTTGTGGTGCTGGGG<br>ACTCACGGGAGACCGCCGGGGTCAACTCGGAGGAAGGTGGGGACGACGT<br>CAAGTCATCATGCCCCTTATGTCTTGGGCTGCACACGTGCTACAATGGCCG<br>GTACAATGAGCTGCGATACCGCGAGGTGGAGCGAATCTCAAAAAGCCGGT<br>CTCAGTTCGGATTGGGGTCTGCAACTCGACCCCATGAAGTCGGAGTCGCTA<br>GTAATCGCAGATCAGCATTGCTGCGGTGAATACGTTCCCGGGCCTTGTACA<br>CACCGCCCGTCACGTCACGAAAGTCGGTAACACCCGAAGCCGGTGGCCCA<br>ACCCCCTTGTGGGGAGGGAGCTGTCGAAGGTGGGACTGGCGATTGGGACG<br>AAGTCGTAAC
